# Supplementary material for: Body Composition and Its Outcomes and Management in Multiple Sclerosis: Narrative Review
Source: Nutrients. 2025 Mar 14;17(6):1021. doi: 10.3390/nu17061021 (PMC11946597; doi:10.3390/nu17061021)
Supplement: Supplementary file 1 [file nutrients-17-01021-s001.zip › nutrients-3465648-supplementary.pdf]

Supplemental Materials

Table S1

Body composition via DEXA and disease- and health-related outcomes in multiple sclerosis (study summary).

Reference	MS Participants	Study Design	Outcome(s)	Results		
				Fat	Bone	Muscle
59	*n* = 33; 81.8% F; mean age: 47 y; 87.9% RRMS	Cross-sectional MS vs non-MS controls	Biomarkers	***Whole-body fat mass & CRP (ρ = 0.39)**<br>***% body fat & CRP (ρ = 0.44)**<br>[X]Trunk fat mass & CRP<br>[X]% trunk fat & CRP<br>*[Results are adjusted for age, sex, and physical activity levels]*		
61	*n* = 40; 0% F; mean age: 51.2 y; 17.5% RRMS	Cross-sectional men with MS with decreased BMD	Biomarkers		[X]Femoral neck BMD & vitamin D, Ca, PHT, SHBG, and T<br>[X]Lumbar spine BMD & vitamin D, Ca, PHT, SHBG and T	
62	*n* = 119; 60.5% F; mean age: 39.2 y; 100% RRMS	Cross-sectional RRMS	Biomarkers		[X]Femoral neck BMD & vitamin D<br>[X]Lumbar spine BMD & vitamin D	
63	*n* = 30; 60% F; mean age: 29.2 y; 100% newly diagnosed	Cross-sectional newly diagnosed MS	Biomarkers		[X]Femoral neck BMD & vitamin D<br>[X]Lumbar spine BMD & vitamin D	
67	*n* = 60; 73.3% F; mean age: 52.5 y; 76.7% RRMS	Cross-sectional MS	Cognition	[X]Whole-body fat mass & BVMT-R, CVLT-2, and SDMT<br>[X]% body fat & BVMT-R, CVLT-2, and SDMT<br>*[Results are adjusted for age and disability status]*	[X]Whole-body BMD & BVMT-R, CVLT-2, and SDMT<br>*[Results are adjusted for age and disability status]*	[X]Whole-body LSTM & BVMT-R, CVLT-2, and SDMT<br>*[Results are adjusted for age and disability status]*
68	*n* = 56; 75% F; mean age: 43.5 y; 89.3% RRMS	Cross-sectional MS with CI vs MS without CI	Cognition		***Osteopenia/Osteoporosis in CI group (59.3 % vs 24.1%)**<br>***Femur BMD & BVMT-R-DR (*r* = 0.39) and -TL (*r* = 0.41), JLO (*r* = 0.34) in both groups combined, and in CI group (*d* = –0.68)**<br>[X]Femur BMD & COWAT, CVLT-2-DR and -TL, DKFS-CS and -DS, PASAT 3s, and SDMT in both groups combined<br>[X]Lumbar spine BMD in CI group<br>*[Results for BVMT-R-DR and -TL, COWAT, CVLT-DR and -TL, DKFS-CS and -DS, JLO, PASAT 3s, and SDMT are adjusted for disability status]*	
69	*n* = 47; 70.2% F; mean age: 50.7 y; 100% RRMS	Cross-sectional RRMS	Cognition	[X]Whole-body fat mass & SDMT<br>[X]% body fat & SDMT<br>*[Results are adjusted for age, sex, disability status, and disease duration]*	[X]Whole-body BMD & SDMT<br>[X]Whole-body BMC & SDMT<br>*[Results are adjusted for age, sex, disability status, and disease duration]*	***Whole-body LSTM & SDMT (ρ = 0.39)**<br>*[Results are adjusted for age, sex, disability status, and disease duration]*

			Mobility	[X]Whole-body fat mass & T25FW and 6MW<br>[X]% body fat & T25FW and 6MW<br>*[Results are adjusted for age, sex, disability status, and disease duration]*	[X]Whole-body BMD & T25FW and 6MW<br>[X]Whole-body BMC & T25FW and 6MW<br>*[Results are adjusted for age, sex, disability status, and disease duration]*	[X]Whole-body LSTM & T25 FW and 6MW<br>*[Results are adjusted for age, sex, disability status, and disease duration]*
			Symptoms	***Whole-body fat mass & MPQ (ρ = 0.32)**<br>[X]Whole-body fat mass & HADS-A and -D, and MFIS<br>[X]% body fat & HADS-A and -D, MFIS, and MPQ<br>*[Results are adjusted for age, sex, disability status, and disease duration]*	[X]Whole-body BMD & HADS-A and -D, MFIS, and MPQ<br>[X]Whole-body BMC & HADS-A and -D, MFIS, and MPQ<br>*[Results are adjusted for age, sex, disability status, and disease duration]*	[X]Whole-body LSTM & HADS-A and -D, MFIS, and MPQ<br>*[Results are adjusted for age, sex, disability status, and disease duration]*
			Fitness	***Whole-body fat mass & VO_{2peak} (ρ = –0.52)**<br>[X]Whole-body fat mass & grip strength, LE_{peak}, and LF_{peak}<br>***% body fat & VO_{2peak} (ρ = –0.56)**<br>[X]% body fat & grip strength, LE_{peak}, and LF_{peak}<br>*[Results are adjusted for age, sex, disability status, and disease duration]*	***Whole-body BMD & LE_{peak} (ρ = 0.35)**<br>[X]Whole-body BMD & grip strength, LF_{peak}, and VO_{2peak}<br>***Whole-body BMC & grip strength (ρ = 0.53), LE_{peak} (ρ = 0.69), LF_{peak} (ρ = 0.50)**<br>[X]Whole-body BMC & VO_{2peak}<br>*[Results are adjusted for age, sex, disability status, and disease duration]*	***Whole-body LSTM & grip strength (ρ = 0.38), LE_{peak} (ρ = 0.48), LF_{peak} (ρ = 0.38)**<br>[X]Whole-body LSTM & VO_{2peak}<br>*[Results are adjusted for age, sex, disability status, and disease duration]*
			Quality of Life	[X]Whole-body fat mass & MSIS-29 physical and psychological HRQOL<br>***% body fat & MSIS-29 psychological HRQOL (ρ = 0.34)**<br>[X]% body fat & MSIS-29 physical HRQOL<br>*[Results are adjusted for age, sex, disability status, and disease duration]*	[X]Whole-body BMD & MSIS-29 physical and psychological HRQOL<br>[X]Whole-body BMC & MSIS-29 physical and psychological HRQOL<br>*[Results are adjusted for age, sex, disability status, and disease duration]*	[X]Whole-body LSTM & MSIS-29 physical and psychological HRQOL<br>*[Results are adjusted for age, sex, disability status, and disease duration]*
78	*n* = 74; 81.1% F; mean age: 47.2 y; 94.6% RRMS	Cross-sectional MS	Mobility	***% body fat & TUG (ρ = 0.35), T25FW (ρ = 0.45), and 6MW (ρ = –0.44)**<br>*[Results are adjusted for age and disease duration]*		
79	*n* = 25; 100% F; mean age: 48.1 y; 84% RRMS	Cross-sectional women with MS vs non-MS controls	Mobility	***% body fat & TUG (*r* = 0.51)**<br>[X]% body fat & SEBT and 6MW		***Leg LSTM & 6MW (*r* = 0.45)**<br>[X]Leg LSTM & TUG and SEBT<br>***Leg LSTM to body mass ratio & TUG (*r* = –0.53) and 6MW (*r* = 0.53)**<br>[X]Leg LSTM to body mass ratio & SEBT
80	*n* = 63; 73% F; mean age: 46.7 y; 92% RRMS	Cross-sectional MS	Mobility	***% body fat and oxygen cost of walking (ρ = –0.37)**<br>*[Results are adjusted for age, sex, disability status, step length, and cadence]*	***Whole-body BMD & oxygen cost of walking (ρ = –0.37)**<br>[X]Whole-body BMC & walking efficiency<br>*[Results are adjusted for age, sex, disability status, step length, and cadence]*	[X]Whole-body LSTM & oxygen cost of walking<br>[X]Weight to LSTM ratio & oxygen cost of walking<br>*[Results are adjusted for age, sex, disability status, step length, and cadence]*
81	*n* = 64; 73.4% F; mean age: 46.7 y; 90.6% RRMS	Cross-sectional MS	Mobility	[X]Whole-body fat mass & MSWS-12, TUG, and 6MW<br>[X]% body fat & MSWS-12, TUG, and 6MW	[X]Whole-body BMD & MSWS-12, TUG, and 6MW<br>[X]Whole-body BMC & MSWS-12, TUG, and 6MW	[X]Whole-body LSTM & MSWS-12, TUG, and 6MW

86	*n* = 110; 76.4% F; mean age: 43.1 y; 90% RRMS	Cross-sectional MS with depression and without depression	Symptoms	^{X}Whole-body fat mass in depression group<br>***% body fat in depression group (OR = 1.07)**<br>^{X}VFL in depression group		^{X}Whole-body LSTM in depression group<br>^{X}PMM in depression group
87	*n* = 77; 74% F; mean age: 49 y; 77.9% RRMS	Cross-sectional MS	Symptoms	^{X}Whole-body and site-specific (trunk, left and right arm, and left and right leg) % body fat & FSS, HADS-A and -D, MFIS, and MPQ<br>*[Results are adjusted for age, sex, and disability status]*	^{X}Whole-body and site-specific (trunk, left and right arm, and left leg) BMD & FSS, HADS-A and -D, MFIS, and MPQ<br>^{X}Whole-body and site-specific (trunk, left and right arm, and left leg) BMC & FSS, HADS-A and -D, MFIS, and MPQ<br>*[Results are adjusted for age, sex, and disability status]*	^{X}Whole-body and site-specific (trunk, left and right arm, and left leg) % LSTM & FSS, HADS-A and -D, MFIS, and MPQ<br>*[Results are adjusted for age, sex, and disability status]*
97	*n* = 51; 88.2% F; mean age: 36 y; 100% RRMS	Cross-sectional MS vs non-MS controls	Quality of Life		***Femur BMD & MSIS-29 physical and psychological HRQOL (NR)**<br>***Lumbar spine BMD & MSIS-29 physical HRQOL (NR)**<br>^{X}Lumbar spine BMD & MSIS-29 psychological HRQOL	

Note: ***** = **Significant relationship**; ^{X} = Nonsignificant relationship; CI = Cognitive impairment; BMC = Bone mineral content; BMD = Bone mineral density; F = Female; LSTM = Lean soft tissue mass MS = Multiple sclerosis; RRMS = Relapsing remitting multiple sclerosis; y = years; Ca = Calcium; CRP = C-reactive protein; NR = No correlation coefficient reported; PHT = Parathyroid hormone; PMM = Predictive muscle mass; SHBG = Sex hormone binding globulin; T = Testosterone; VFL = Visceral fat level; BVMT-R, -DR, -TL = Brief Visuospatial Memory Test – Revised, - Delayed Recall, - Total Learning; COWAT = Controlled Oral Word Association Test; CVLT-2,-DR, -TL = California Verbal Learning Test – Second Edition, -Delayed Recall, -Total Learning; DKFS, -CS, -DS = Delis-Kaplan Executive Function System Sorting Test, - Correct Sorts, - Description Score; JLO = Judgment of Line Orientation Test; PASAT 3s = Paced Auditory Serial Addition Test 3.0s; SDMT = Symbol Digit Modalities Test; MSWS-12 = 12-Item Multiple Sclerosis Walking Scale; SEBT = Star Excursion Balance Test; TUG = Timed Up and Go; T25FW = Timed 25-Foot Walk; 6MW = Six-Minute Walk; FSS = Fatigue Severity Scale; MSIS-29 = 29-Item Multiple Sclerosis Impact Scale; HADS,-A, -D, Hospital Anxiety and Depression Scale, -Anxiety, -Depression; MFIS = Modified Fatigue Impact Scale; MPQ = McGill Pain Questionnaire; LE_{peak} = Peak torque of knee extensors; LF_{peak} = Peak torque of knee flexors; VO_{2peak} = Peak oxygen uptake; HRQOL = Health related quality of life.

Table S2

Body composition via DEXA and interventions/management in multiple sclerosis (study summary).

Reference	Participants	Duration	Intervention(s)	Results: Fat	Results: Bone	Results: Muscle
105	**MS**			^{X}Whole-body fat mass Δ (pre-post) in MS and non-MS controls<br>^{X}Whole-body fat mass Δ (pre-post) MS vs non-MS controls<br>^{X}% body fat Δ (pre-post) in MS and non-MS controls<br>^{X}% body fat Δ (pre-post) in MS vs non-MS controls		^{X}Whole-body LSTM Δ (pre-post) in MS and non-MS controls<br>^{X}Whole-body LSTM Δ (pre-post) MS vs non-MS controls
	n = 18; 33.3% F; mean age: 41.7 y; mild disability	6 months	Home-based periodized HIIT bicycle training intervention			
	**Non-MS Controls**					
	n = 19; 26.3% F; mean age: 41.5 y	6 months	Home-based periodized HIIT bicycle training intervention			
106	**MS PER**			^{X}Whole-body fat mass Δ (pre-post) in MS PER<br>***Whole-body fat mass Δ (pre-post) in MS CLA (Δ = –3%, *p* = 0.002)**<br>***Whole-body fat mass Δ (pre-post) MS PER (–0.1 kg) vs MS CLA (–0.7 kg), (*p* = 0.047)**<br>^{X}% body fat Δ (pre-post) in MS PER<br>***% body fat Δ (pre-post) in MS CLA (Δ = –2%, *p* = 0.005)**<br>^{X}% body fat Δ (pre-post) MS PER vs MS CLA		^{X}Whole-body LSTM Δ (pre-post) in MS PER and MS CLA<br>^{X}Whole-body LSTM Δ (pre-post) MS PER vs MS CLA
	n = 15; 60% F; mean age: 41 y; mild disability	12 weeks	Periodized HIIT bicycle training program			
	**MS CLA**					
	n = 13; 61.5% F; mean age: 43 y; mild disability	12 weeks	Classic endurance bicycle training program (i.e., low-to-moderate intensity)			
107	**HCTR**			***% body fat Δ (pre-post) in HCTR (Δ = –2.5%, *p* = 0.02)**<br>***% body fat Δ (pre-post) in HITR (Δ = –3.9%, *p* = 0.04)**<br>^{X}% body fat Δ (pre-post) in SED<br>^{X}% body fat Δ (pre-post) SED vs HCTR<br>^{X}% body fat Δ (pre-post) SED vs HITR		^{X}Whole-body LSTM Δ (pre-post) in HCTR<br>***Whole-body LSTM Δ (pre-post) in HITR (Δ = 1.4%, *p* = 0.01)**<br>^{X}Whole-body LSTM Δ (pre-post) in SED<br>^{X}Whole-body LSTM Δ (pre-post) SED vs HCTR<br>^{X}Whole-body LSTM Δ (pre-post) SED vs HITR
	n = 11; 54.5% F; mean age: 47 y; mild disability	12 weeks	High intensity continuous cardiovascular cycle ergometer training plus resistance training			
	**HITR**					
	n = 12; 58.3% F; mean age: 43 y; mild disability	12 weeks	High intensity interval cycle ergometer training group plus resistance training			
	**SED**					
	n = 11; 81.8% F; mean age: 47 y; mild disability	12 weeks	Sedentary control group (i.e., no change to routine)			

108	MS			^{x}% body fat Δ (pre-post) in MS<br>^{x}% body fat Δ (pre-post) in non-MS controls<br>^{x}% body fat Δ (pre-post) MS vs non-MS controls		^{X}Whole-body LSTM Δ (pre-post) in MS<br>^{X}Whole-body LSTM Δ (pre-post) in non-MS controls<br>^{X}Whole-body LSTM Δ (pre-post) MS vs non-MS controls
	n = 29; 86.2% F; mean age: 41.3 y; mild disability	10 months	Home-based structured running program (i.e., moderate-to-high intensity)			
	Non-MS Controls					
	n = 26; 65.4% F; mean age: 41.2 y; no MS; mild disability	10 months	Home-based structured running program (i.e., moderate-to-high intensity)			
110	Intervention			^{X}Whole-body fat mass Δ (post) Intervention vs Waitlist<br>^{x}% body fat Δ (post) Intervention vs Waitlist	***Whole-body BMD Δ (post) Intervention vs Waitlist ($F_{1,69}$ = 6.56, p = 0.01) [not significant using adjusted critical value (*p* < 0.008)]**<br>***Whole-body BMC Δ (post) Intervention vs Waitlist ($F_{1,69}$ = 4.30, p = 0.04) [not significant using adjusted critical value (*p* < 0.008)]**	^{X}Whole-body LSTM Δ (post) Intervention vs Waitlist
	n = 41; 73.1% F; mean age: 48.4 y; ambulatory	6 months	Theory-based internet-delivered behavioral intervention to promote lifestyle physical activity (i.e., walking)			
	Waitlist					
	n = 41; 78.0% F; mean age: 49.5 y; ambulatory	6 months	Waitlist control (i.e., no change to routine)			
112	Pilates			^{X}Whole-body fat mass Δ (pre-post) Pilates vs No Pilates<br>^{x}% body fat Δ (pre-post) Pilates vs No Pilates		^{X}Whole-body LSTM Δ (pre-post) Pilates vs No Pilates
	n = 15; 80% F; mean age: 45.7 y; not restricted to a wheelchair or scooter	12 weeks	Pilates two-times/ week plus one weekly massage therapy session			
	No Pilates					
	n = 15; 73.3% F; mean age: 45.1 y; not restricted to a wheelchair or scooter	12 weeks	One weekly massage therapy session			
124	Intervention			***% body fat Δ (pre-post) Intervention (–3.4%) vs TAU (–0.4%), (*p* = 0.001)**		
	n = 34; 82.4% F; mean age: 46.0 y; 94.1%	6 months	Theory-based group telehealth and home-based program to gradually increase physical activity to achieve a total of at least 150 minutes of moderate intensity			

			physical activity five days/week<br>**PLUS**<br>Theory-based group telehealth and home-based program to consume a reduced calorie diet (i.e., 1200-1500 calories/day with composition guidelines)			
	TAU					
	n = 30; 83.3% F; mean age: 47.5 y; 100%	6 months	Waitlist TAU (i.e., were sent content regarding healthy eating and obesity prevention)			
114	IF-5:2			***Whole-body fat mass Δ (weekly) IF-5:2 (–119.8 grams) vs CR-78% (–330.1 grams), (*p* = 0.03)**<br>[X]Whole-body fat mass Δ (weekly) IF-5:2 vs NR-100%<br>[X]Whole-body fat mass Δ (weekly) CR-78% vs NR-100%<br>[X]% body fat Δ (weekly) IF-5:2 vs CR-78%<br>[X]% body fat Δ (weekly) IF-5:2 vs NR-100%<br>[X]% body fat Δ (weekly) CR-78% vs NR-100%<br>[X]VAT mass Δ (weekly) IF-5:2 vs CR-78%<br>[X]VAT mass Δ (weekly) IF-5:2 vs NR-100%<br>[X]VAT mass Δ (weekly) CR-78% vs NR-100%<br>[X]% VAT Δ (weekly) IF-5:2 vs CR-78%<br>[X]% VAT Δ (weekly) IF-5:2 vs NR-100%<br>[X]% VAT Δ (weekly) CR-78% vs NR-100%		[X]Whole-body LSTM Δ (weekly) IF-5:2 vs CR-78%<br>[X]Whole-body LSTM Δ (weekly) IF-5:2 vs NR-100%<br>[X]Whole-body LSTM Δ (weekly) CR-78% vs NR-100%
	n = 12; 83.3% F; mean age: 38.5 y; 100% RRMS	8 weeks	Controlled feeding IF-5:2 (i.e., 100% daily caloric needs five days/week and 25% daily caloric needs two consecutive days/week)			
	CR-78%					
	n = 12; 83.3% F; mean age: 40.5 y; 100% RRMS	8 weeks	Controlled feeding calorie restriction (i.e., 78% daily caloric needs seven days/week)			
	NR-100%					
	n = 12; 75% F; mean age: 33.3 y; 100% RRMS	8 weeks	Control with no calorie restriction (i.e., 100% daily caloric needs seven days/week)			

115	iCR			***Whole-body fat mass Δ (pre-post) in iCR (Δ = –2.21 g, *p* < 0.0001)**<br>^{X}Whole-body fat mass Δ (pre-post) in NR<br>^{X}Whole-body fat mass Δ (pre-post) iCR vs NR<br>***Trunk fat mass Δ (pre-post) in iCR (Δ = –1.4 g, *p* < 0.0001)**<br>^{X}Trunk fat mass Δ (pre-post) in NR<br>^{X}Trunk fat mass Δ (pre-post) iCR vs NR		^{X}Whole-body LSTM Δ (pre-post) in iCR<br>^{X}Whole-body LSTM Δ (pre-post) in NR<br>^{X}Whole-body LSTM Δ (pre-post) iCR vs NR
	n = 22; 90.9% F; mean age: 50 y; 100% RRMS	12 weeks	IF-5:2 diet (i.e., 500 calorie limit two non-consecutive days/week and usual intake with instructions to monitor intake/not overeat for five days)			
	NR					
	n = 20; 80% F; mean age: 46.4 y; 100% RRMS	12 weeks	No restriction/no change to routine			
116	8-h TRE			^{X}% body fat Δ (pre-post)		^{X}% LSTM Δ (pre-post)
	n = 12; 83.3% F; mean age: 46 y; 100% RRMS	8 weeks	Intermittent fasting (TRE) – 8 hour eating window/fast for 16 hours			
117	Low GI			***Whole-body fat mass Δ (pre-post) (Δ = –1.94 kg, *p* = 0.006)**		***Whole-body LSTM Δ (pre-post) (Δ = –0.72 kg, *p* = 0.035)**
	n = 20; 85% F; mean age: 46.15 y; 100% RRMS	12 weeks	Theory-based behavioral intervention to eat low glycemic index diet			

Note: *** = Significant relationship**; ^{X} = No significant relationship; Δ = Change/difference; BMC = Bone mineral content; BMD = Bone mineral density; C = Non-multiple sclerosis controls; F = Female; GI = Glycemic index; g = Grams; HIIT = High-intensity interval exercise therapy; IF = Intermittent fasting; kg = Kilograms; LSTM = Lean soft tissue mass; MS = Multiple sclerosis; RRMS = Relapsing-remitting multiple sclerosis; TAU = Treatment as usual; TRE = Time restricted eating; y = years; VAT = Visceral adipose tissue.
